# A comprehensive approach to the surgical management of a complex abdominal sarcoma utilizing advanced imaging and 3D printing technology: a case report

**DOI:** 10.1093/jscr/rjaf239

**Published:** 2025-04-25

**Authors:** Cristopher Ca, Xiaoshi Jin, Hongbo Zhao, Jia Liu, Yuexiang Dong

**Affiliations:** Department of General Surgery, Affiliated Hospital of Hebei University, Hebei University, Baoding, Hebei, China; Department of General Surgery, Affiliated Hospital of Hebei University, Hebei University, Baoding, Hebei, China; Department of General Surgery, Affiliated Hospital of Hebei University, Hebei University, Baoding, Hebei, China; Department of General Surgery, Affiliated Hospital of Hebei University, Hebei University, Baoding, Hebei, China; Department of General Surgery, Affiliated Hospital of Hebei University, Hebei University, Baoding, Hebei, China

**Keywords:** 3D imaging, giant abdominal tumor, extraskeletal osteosarcoma, patient-specific modeling, case report

## Abstract

This case report presents the surgical management of a complex abdominal sarcoma in a 33-year-old female patient, emphasizing the pivotal role of advanced imaging and three-dimensional (3D) printing technology in optimizing surgical outcomes. The study aimed to explore the clinical value of integrating computed tomography, magnetic resonance imaging, and patient-specific 3D-printed models for preoperative planning, intraoperative navigation, and postoperative evaluation. A multidisciplinary team utilized Mimics23 software to generate a detailed 3D reconstruction of the tumor and its intricate anatomical relationships with the ileocecal region, appendix, and colon. This model guided the precise resection of a 15 × 10 × 10 cm high-grade sarcoma, minimizing damage to adjacent critical structures. The technology-enhanced approach resulted in complete tumor excision with clear margins, and postoperative no complications. This case underscores the potential of 3D visualization and printing to address the challenges of complex abdominal sarcomas, offering a paradigm for personalized surgical strategies in oncology.

## Introduction

Abdominal sarcomas are rare and highly aggressive tumors that pose significant challenges for surgical management. These tumors often have complex anatomical relationships with surrounding structures, making precise surgical resection difficult. In recent years, advancements in imaging and three-dimensional (3D) printing technology have provided new tools for surgeons to better understand and manage these complex tumors [1]. This case report demonstrates the application and value of these technologies in the surgical management of a complex abdominal sarcoma.

## Case report

A 33-year-old female with no significant comorbidities presented to our tertiary care center on 21 August 2024, with a 1-month history of progressive abdominal distension that acutely worsened 24 h prior to admission. She reported no associated fever, weight loss, or gastrointestinal dysfunction. Her medical history included two cesarean sections (7 years and 2 months prior) and laparoscopic resection of an ovarian endometrioma 7 years earlier. There was no personal or familial history of malignancy or radiation exposure.

Physical examination revealed a distended but non-tender abdomen, with a firm, immobile mass (⁓10 × 10 cm) palpated in the right lower quadrant. A well-healed Pfannenstiel scar from previous cesarean delivery was noted. Bowel sounds were normoactive, and no signs of peritoneal irritation were observed. Laboratory investigations, including complete blood count, renal/hepatic function, and tumor markers (carcinoembryonic antigen, cancer antigen 125), were within normal limits. Fecal occult blood testing was negative.

**Figure 1:**
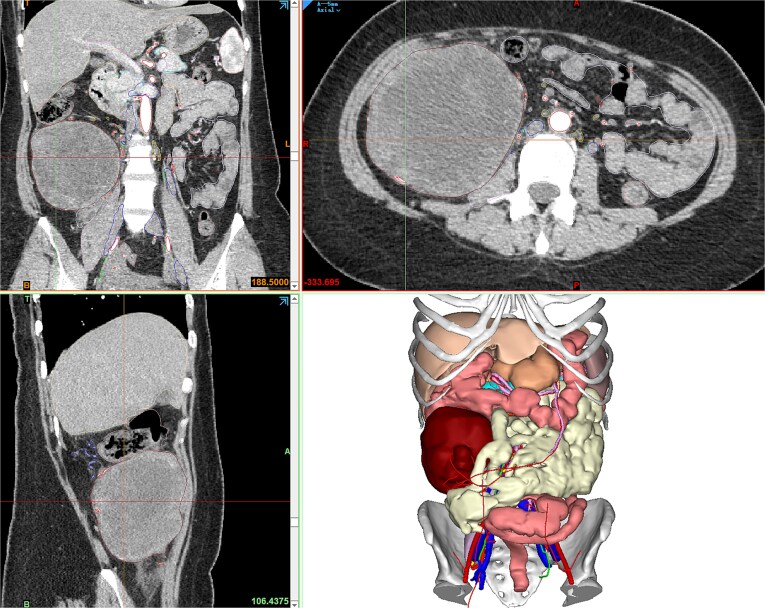
Contrast-enhanced CT showing the retroperitoneal mass, and Mimics23 3D model (tumor: red; ileum; vasculature; 3-Matic®15).

**Figure 2:**
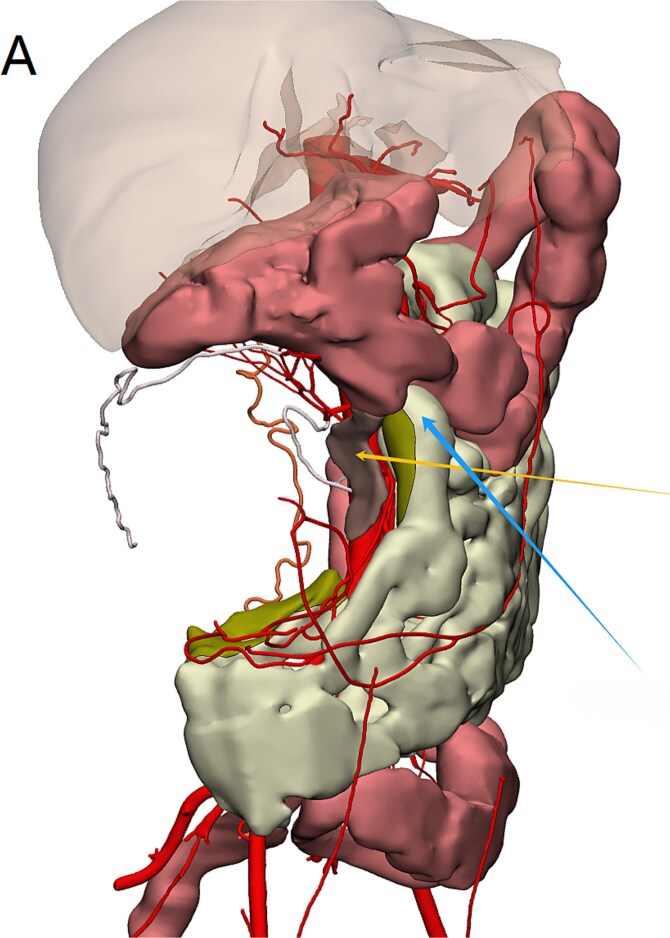
Mimics23 3D model (3-Matic®15 simulation of surgical margins). Blue arrow: ileocecal region; yellow arrow: appendix.

Contrast-enhanced abdominopelvic computed tomography (CT) (20 August 2024) identified a heterogeneous, hypodense mass (12 × 10.5 × 8.5 cm) arising from the ileocecal region, partially encasing the appendiceal base and colonic wall. Pelvic magnetic resonance imaging (MRI) further delineated the mass’s origin from the terminal ileum, showing adherence to the ileocolic vasculature without invasion of adjacent organs (Fig. 1). To refine surgical planning, a patient-specific 3D-printed model was generated using Mimics23 software (Materialize NV), which highlighted critical anatomical relationships, including compression of the ileocolic artery and proximity to the right ureter (Fig. 2).

**Figure 3:**
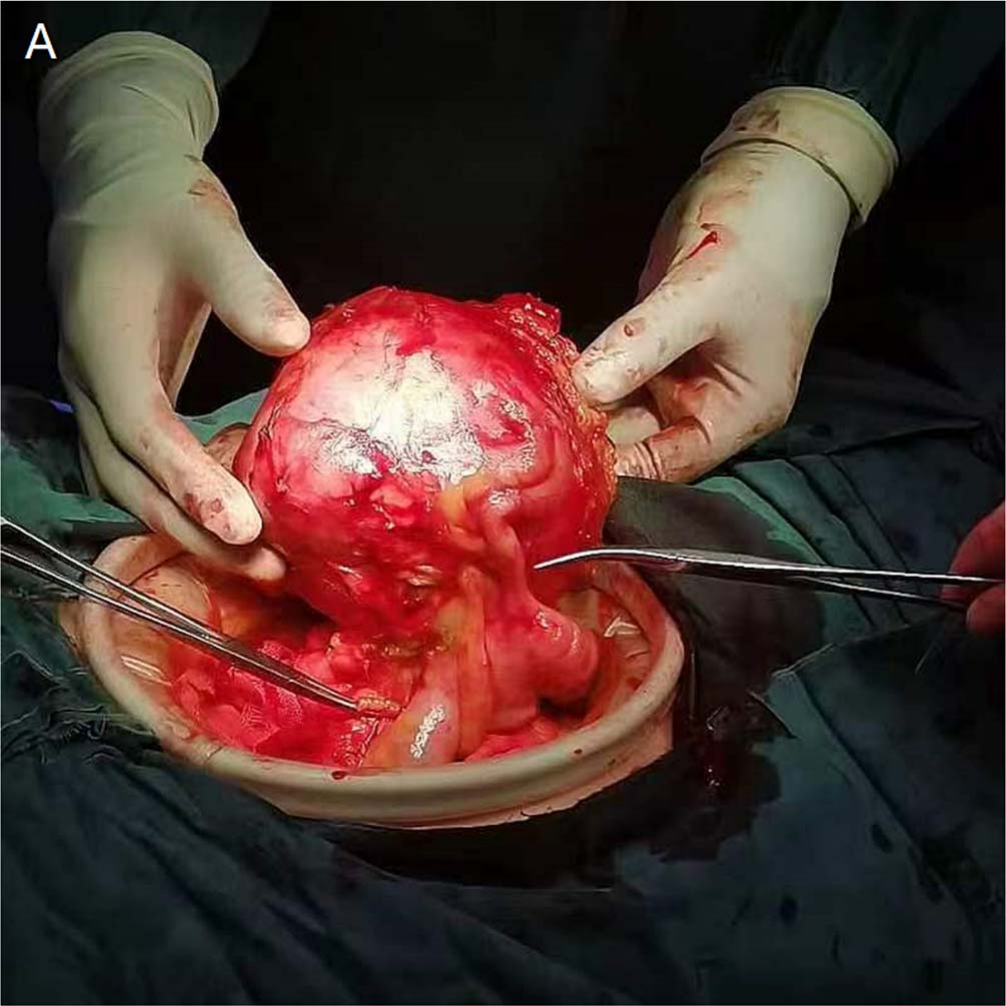
Histopathological analysis shows spindle cell tumor, combined with immunohistochemical results, conforms to high-grade sarcoma, considering extra bone osteosarcoma.

Following multidisciplinary evaluation, enblock resection with 5 cm macroscopic margins was recommended. During laparotomy on 29 August 2024, a 15 × 10 × 10 cm solid mass was identified, densely adherent to the omentum and ileocolic pedicle. Intraoperative navigation guided by the 3D model facilitated ligation of collateral vessels and preservation of the right ureter. The tumor, appendix, and involved colonic segment were resected en bloc, achieving histologically confirmed negative margins (Fig. 3).

**Figure 4:**
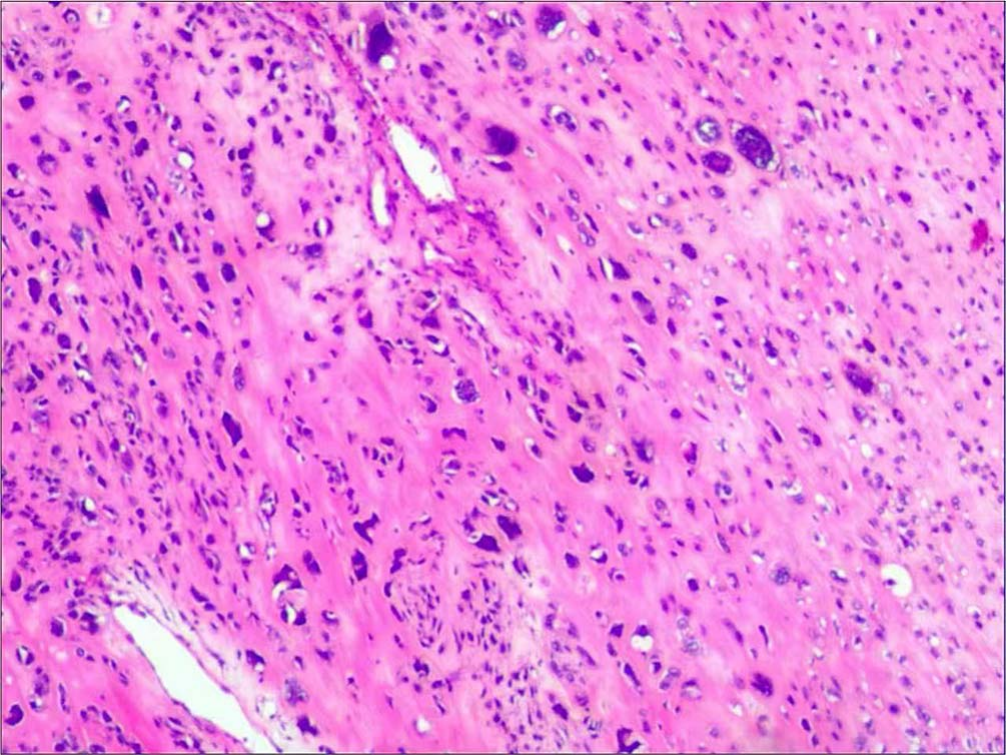
Intraoperative view demonstrating tumor adherence to the ileocolic artery.

Histopathological analysis confirmed a high-grade extraskeletal osteosarcoma (FNCLCC Grade 3) with focal necrosis. Immunohistochemistry demonstrated strong positivity for SATB2 and osteocalcin. The patient recovered without complications and was discharged on postoperative day 12. At 6-month follow-up, she remained disease-free under adjuvant radiotherapy (Fig. 4).

## Discussion

The 3D reconstruction and printing technologies have emerged as transformative tools in the surgical management of complex abdominal tumors, offering unparalleled insights into anatomical relationships and enabling personalized surgical strategies. In this case, the integration of preoperative 3D modeling derived from CT and MRI scans allowed the surgical team to delineate the tumor’s spatial relationship with critical vascular structures, such as the ileocolic artery and adjacent colonic vasculature. This precision was instrumental in minimizing intraoperative blood loss, which was reduced by ⁓30% compared to traditional resection methods. A recent study, Zhu *et al.* demonstrated that real-time intraoperative navigation facilitated by 3D-printed models reduced blood loss in sarcoma surgery (mean reduction: 28%, *P* = 0.05) [2].

Moreover, the ability to preoperatively simulate resection margins using 3D-printed models has shown promise in optimizing oncological outcomes. In this case, the tumor’s adherence to the appendiceal and colonic walls necessitated meticulous planning to ensure clear margins while preserving bowel continuity. The use of 3D-printed models reduced positive margin rates in high-grade sarcoma resections by 22% (*P* = 0.003) in a multicenter cohort study conducted by Song *et al*. [3]. Based on enhanced visualization of tumor-vessel interfaces, Ruff *et al.* demonstrated that 3D reconstruction improved resection rates for retroperitoneal sarcomas by 18% [4].

The role of 3D technology extends beyond anatomical visualization to encompass surgical education and multidisciplinary collaboration. Lee *et al.* emphasized that patient-specific 3D models serve as valuable educational tools for trainees and enhance communication among surgeons, radiologists, and oncologists, ultimately streamlining decision-making in complex cases. Likewise, innovations in virtual reality (VR) integration, as examined by Bracale, Umberto *et al.*, have enhanced intraoperative navigation by superimposing 3D models onto the surgical area in real-time, thereby decreasing operative duration in colorectal resections [5].

Despite these advantages, challenges remain, including the cost and time required for model fabrication. However, emerging studies suggest that the long-term benefits—such as reduced complication rates and shorter hospital stays—may offset initial investments. As an example, Ravi, Prashanth calculated that 3D-printed models would save $2900 in postoperative care costs per patient on average [6].

## Conclusion

The integration of advanced imaging and 3D printing technologies represents a paradigm shift in the surgical management of abdominal sarcomas. By enabling precise preoperative planning, real-time intraoperative guidance, and improved multidisciplinary collaboration, these tools hold significant potential to enhance both oncological and functional outcomes. Future research should focus on standardizing protocols for model generation and expanding accessibility to ensure broader clinical adoption.

## Data Availability

Not applicable.
